# The Role of Etanercept in Controlling Clinical and Radiological Progression in Rheumatoid Arthritis: A Systematic Review

**DOI:** 10.7759/cureus.58112

**Published:** 2024-04-12

**Authors:** Samrah Ejaz, Simhachalam Gurugubelli, Suviksh K Prathi, Yaneisi Palou Martinez, Divine Besong Arrey Agbor, Priyanka Panday, Ann Kashmer Yu

**Affiliations:** 1 Internal Medicine, California Institute of Behavioral Neurosciences and Psychology, Fairfield, USA; 2 Internal Medicine, Memorial Healthcare, Gulfport, USA; 3 General Medicine, California Institute of Behavioral Neurosciences and Psychology, Fairfield, USA; 4 General Medicine, St. George's University School of Medicine, St. Georges, GRD; 5 Medicine, California Institute of Behavioral Neurosciences and Psychology, Fairfield, USA; 6 Clinical Research and Internal Medicine, California Institute of Behavioral Neurosciences and Psychology, Fairfield, USA; 7 Internal Medicine, Richmond University Medical Center, New York City, USA; 8 Research, California Institute of Behavioral Neurosciences and Psychology, Fairfield, USA

**Keywords:** anti-tnf, dmards, etanercept, methotrexate, rheumatoid arthritis

## Abstract

Etanercept (ETN) is a disease-modifying anti-rheumatic drug (DMARD) used in the treatment of rheumatoid arthritis (RA) that works as a tumor necrosis factor inhibitor (TNF inhibitor) by blocking the effects of naturally occurring TNF. This review will evaluate the effect of ETN as a monotherapy or combination therapy with methotrexate (MTX) in the treatment of RA. This systematic review was carried out in accordance with the Preferred Reporting Items for Systematic Reviews and Meta-Analysis (PRISMA) 2020 guidelines. A systematic search was done on PubMed and Google Scholar from 1999 to 2023. Predefined eligibility criteria were set for selected studies, which include: free full-text articles published; randomized control trials (RCTs); systematic reviews and meta-analyses; and observational studies in a patient with RA treated with ETN as initial therapy or as an add-on to conventional disease-modified therapy. Hence, the data had been extracted, and a quality assessment of each study was done by two individual authors. When comparing patients who received 15-25 mg of MTX with those who also received 25 mg of ETN in combination, 71% achieved American College of Rheumatology 20 (ACR20) by 24 weeks, compared to 27% in the MTX and placebo groups (p<0.001), and 39% achieved American College of Rheumatology 50 (ACR50), compared to 3% in the placebo + MTX group (p<0.001). Low disease activity (DAS 28) was more common in patients who had both MTX and ETN (64.5% with DAS <2.4 and 56.3% with DAS 28 <3.2) compared to patients who received only one medication (44.4% with DAS <2.4 and 33.2% with DAS 28 <3.2 for ETN and 38.6% with DAS <2.4 and 28.5% with DAS 28 <3.2 for MTX, with P<0.01). ETN demonstrated smaller changes from baseline in the modified Sharp score (TSS) and erosion scores (ES) at 12 months and two years, as well as a decreased change in the ES score at one year (with a trend of P value = 0.06 for the TSS score), in comparison to those receiving DMARD. Reactions at the injection site (42% vs. 7%, P<0.001) were the only events that occurred significantly more frequently in the ETN plus-MTX group. Combining ETN and MTX appears to help control RA symptoms by decreasing the American College of Rheumatology (ACR) response and DAS score, as well as halting the disease's progression on X-rays. The most common adverse effects were reactions to ETN administered alone at the injection site, likely because of patient awareness of the treatment received. There was also concern about tuberculosis and malignancy, but no recent data is available. Therefore, a larger clinical trial with longer follow-up is required to ascertain long-term safety and benefits.

## Introduction and background

Rheumatoid arthritis (RA) is a chronic autoimmune disease that primarily affects hands and feet symmetrically, but any synovial joint can be affected and can eventually result in joint destruction [1-3]. It is characterized by inflammation, pain, and swelling in the joints, which can lead to long-term complications if left untreated [1-3].

RA is a prevalent illness that affects one percent of people in the UK. With a peak onset between the ages of 30 and 50, the incidence rises with age. Women are twice as likely as men to have RA [1,4]. According to the WHO, 18 million people worldwide suffered from RA in 2019, of whom 70% were women, and 55% of those affected were over 55 [4].

The use of conventional disease-modifying anti-rheumatic medications (cDMARDs) remains the first-line treatment for RA, which can cause a remission of the disease in about half of the patients who take them [4]. It can also be demonstrated to lower disease activity, delay joint deterioration, and enhance quality of life (QoL) [5-10]. Among cDMARDs, methotrexate (MTX) is the most significant and practical drug [5-10]. Although, over time, a lot of these medications show increased toxicity and decreased efficacy [11].

In recent years, there has been increasing recognition of the role that cytokines, particularly tumor necrosis factor (TNF), play in the pathogenesis of RA [12-14]. The advent of anti-TNF therapies has revolutionized the treatment of RA by showing a reduction in symptoms, halting joint deterioration, and improving the QoL [12-15].

Etanercept (ETN) is a soluble TNF receptor fusion protein that binds to and deactivates TNF, thus reducing joint inflammation [16-22]. It is prescribed when one or more cDMARDs fail to control the clinical symptoms of RA [16-22]. Various systematic reviews and meta-analyses have been done in the past to check the efficacy and safety of ETN in patients with RA. This systematic review will assess whether ETN is effective as monotherapy or in combination with MTX in controlling the symptoms of RA, based on all the available data.

## Review

Method

The preferred Reporting Items for Systematic Reviews and Meta-Analysis (PRISMA) 2020 guidelines [23] is used to conduct this Systematic Review.

Eligibility criteria

The published studies were evaluated for inclusion based on the population, intervention, comparison, and outcome (PICO) criteria: Population, patients with early or longstanding RA; Intervention: ETN; Comparison: placebo or MTX; and outcome: improvement in clinical symptoms, radiographic progression, and side effects. Additional inclusion and exclusion criteria were also applied to Inclusion, free full-text articles published, randomized control trials (RCTs), systematic reviews and meta-analyses, and observational studies; Exclusion: non-English literature, animal studies, grey literature, biosimilars in RA.

Databases and search strategy

Electronic databases, including PubMed and Google Search, were used to retrieve studies from 1999 to October 2023. Keywords and the Medical Subject Headings (MeSH) strategy were used for field search using Boolean "OR" and "AND". All citations were collected in Microsoft Excel 2021 in alphabetical order. Firstly, duplicates were removed, and then irrelevant articles were removed by screening through title and abstract. Finally, the full text was retrieved for further analysis. A detailed Medical Subject Headings (MeSH) search strategy can be seen in Table 1.

**Table 1:** A detailed MeSH search strategy. MeSH: Medical Subject Headings; DMARD: disease-modifying anti-rheumatic drugs; ANTI-TNF: anti-tumor necrosis factor

Database	Keywords	MeSH Terms	Filters	Results
PubMed	Rheumatoid arthritis, joint inflammation, arthritis	Rheumatoid Arthritis OR Joint Inflammation OR "Arthritis, Rheumatoid/drug therapy"[Mesh]	-	405,380
	Etanercept, anti-TNF	Etanercept OR Anti-TNF OR "Etanercept/therapeutic use"[Mesh]	-	20,774 results
	Methotrexate, DMARDS	Methotrexate OR DMARDs OR "Methotrexate/therapeutic use"[Mesh]	-	518,288 results
	Rheumatoid arthritis, joint inflammation, arthritis, etanercept, anti-TNF, methotrexate, DMARDs. (Boolean "AND" is used to combine all the MeSH terms)	((Rheumatoid Arthritis OR Joint Inflammation OR "Arthritis, Rheumatoid/drug therapy"[Mesh]) AND (Etanercept OR Anti-TNF OR "Etanercept/therapeutic use"[Mesh])) AND (Methotrexate OR DMARDs OR "Methotrexate/therapeutic use"[Mesh])	-	7,185
		((Rheumatoid Arthritis OR Joint Inflammation OR "Arthritis, Rheumatoid/drug therapy"[Mesh]) AND (Etanercept OR Anti-TNF OR "Etanercept/therapeutic use"[Mesh])) AND (Methotrexate OR DMARDs OR "Methotrexate/therapeutic use"[Mesh]) Filters: Free full text	Free Full-Text, Years 1999-2023	2542

Data collection and analysis

Each study was reviewed to ascertain that inclusion criteria were met in individual studies. From each study, the following items were extracted: First author name, year of publication, study type, intervention studied, participant number, study purpose, outcome, and result.

Outcome

The outcomes measured from different studies are improvement in clinical symptoms using American College of Rheumatology 20, 50, or 70 (ACR20, ACR50, or ACR70), disease activity by measuring the disease activity score (DAS 28), radiographic progression using the modified Sharp score, and adverse events (AEs).

Risk of bias in individual studies

The quality appraisal and risk of bias were done by using tools depending on the study type. Two review authors evaluated each study individually. We use the Cochrane Collaboration Risk of Bias Tool (CCRBT) for RCTs, the Newcastle Ottawa Scale (NOS) for Cohort Studies) and Assessment of Multiple Systematic Reviews 2 (AMSTAR 2) for systematic reviews and meta-analyses [24-26]. Each appraisal tool has distinct criteria and scoring systems. Tools that receive a score of “LOW RISK,” “YES,” “PARTIAL YES,” or “1” are awarded a point. When “2” is displayed, two points are given. For each assessment tool, a minimum score of 70% is considered acceptable (Table 2).

**Table 2:** Quality appraisal of individual studies. RoB: risk of bias; RCTs: randomized control trials; CCRBT: Cochrane Collaboration Risk of Bias Tool; AMSTAR-2: assessment of multiple systematic reviews 2; NOS: Newcastle Ottawa Scale; PICO: population, intervention, comparison, and outcome

Quality Appraisal Tool	Study Type	Domains and Their Characteristics	Total Score	Approved Score (>70%)	Approved Studies
CCRBT [24]	RCTs	Seven domains: Selection bias (random sequence generation, allocation concealment), reporting bias (selective reporting), other sources of bias, performance bias (blinding participants and personnel), detection bias (blinding outcome assessment), attrition bias (incomplete outcome data). Bias assessed as high risk, low risk, or unclear	7	5	Weinblatt et al. 1999 [12], Bathon et al., 2006 [13], Heijde et al. 2007 [27], Emery et al. 2010 [28], Moreland et al. 2012 [29], Gallo et al. 2016 [30]
AMSTAR-2 [26]	Systematic review, Meta-analysis	16 domains: (1) Are PICO components included in the research questions and inclusion criteria for the review? (2) Did the review report explicitly state that methods had been established prior to the review being conducted, and did it provide justification for any significant deviations from the protocol? (3) Explanation of the selection of the study design for inclusion; (4) Comprehensive literature search strategy? (5) Study selection in duplicate? (6) Data extraction in duplicate? (7) List of excluded studies to justify exclusions. (8) Included studies in adequate detail. (9) Satisfactory technique for assessing the risk of bias (RoB) in individual studies that were included in the review? (10) The sources of funding for the studies included in the review. (11) If meta-analysis was performed, did the review authors use appropriate methods for the statistical combination of results? (12) If meta-analysis was performed, did the review authors assess the potential impact of RoB in individual studies on the result of meta-analysis or other evidence synthesis? (13) Account of RoB in individual studies when interpreting or discussing the results of the review? (14) Satisfactory explanation for, and discussion of, any heterogeneity observed in the results of the review? (15) Did the review authors carry out adequate investigation of publication bias (small study bias) and discuss its likely impact on the results of the review if they performed quantitative synthesis? (16) Report any potential sources of conflict of interest, including any funding they received for conducting their review. Assessed as yes, partial yes, or no.	16	12	Blumenauer et al. 2003 [6], Chen et al. 2016 [7], Wu et al. 2021 [31]
NOS [25]	Cohort	Eight domains: (1) Representativeness of the exposed cohort. (2) Selection of the non-exposed cohort. (3) Ascertainment of exposure. (4) Demonstration that outcome of interest was not present at the start of the study. (5) Comparability of cohort on the basis of the design or analysis*. (6) Assessment of outcome. (7) Was follow-up long enough for outcomes to occur? (8) Adequacy of follow-up of cohorts. Scoring was done by giving a point on each domain. Scored as 0,1,2. *Maximum of two points can be given for comparability.	8	6	Wassenberg et al. 2023 [32], Pappas et al. 2023 [33]

Result

Study Selection and Quality Assessment

**Figure 1:**
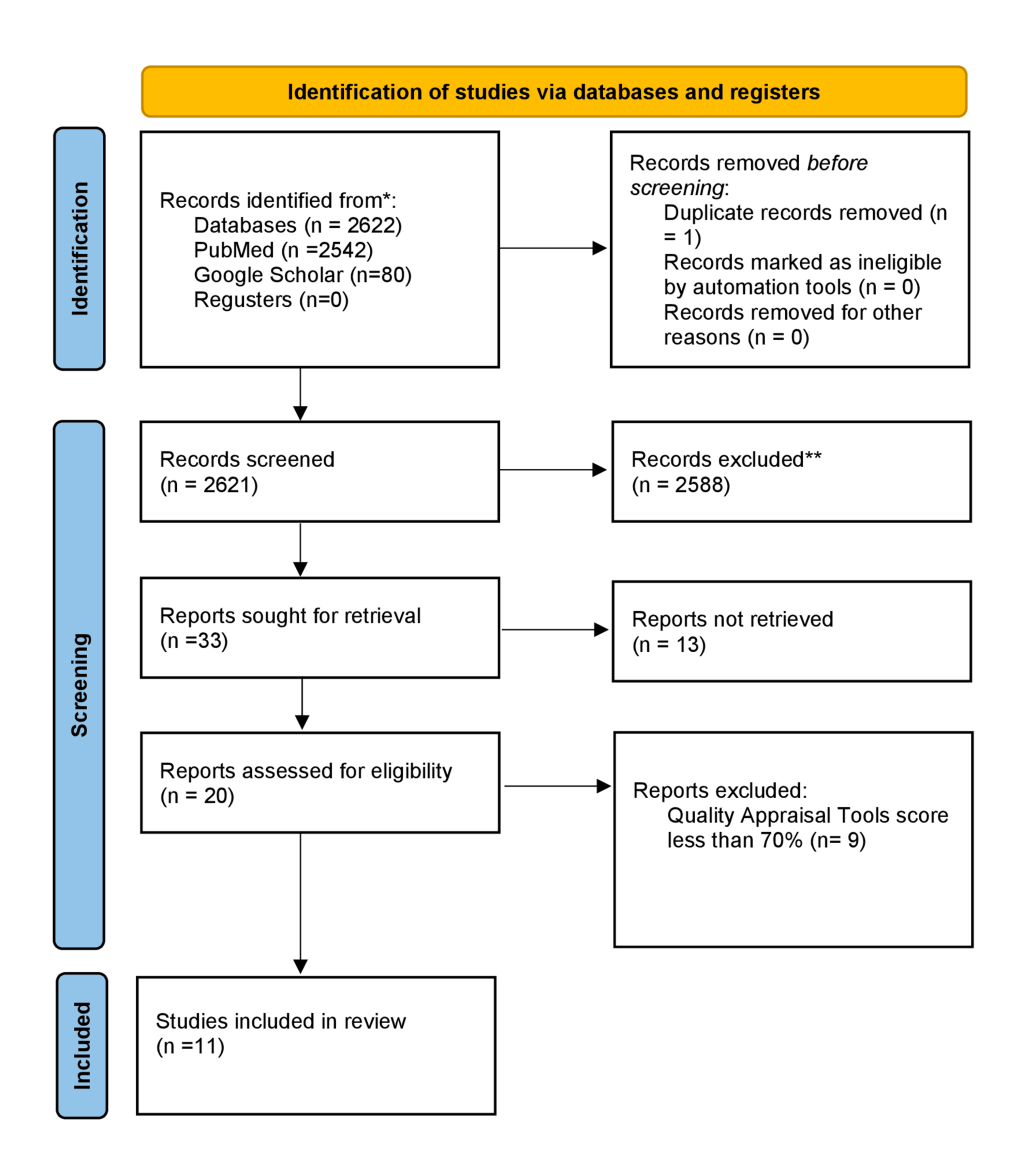
Flowchart of the study search selection CCRBT: Cochrane Collaboration Risk of Bias Tool; AMSTAR 2: Assessment of Multiple Systematic Reviews 2; NOS: Newcastle Ottawa scale The flowchart is created by the authors of this article.

While conducting our systematic review, we adhered to the PRISMA criteria [23]. As shown in Figure 1, we systematically searched multiple electronic databases, such as PubMed and Google Scholar, for data collection. We found 2622 articles in electronic databases. After duplicate removal, we excluded 2588 articles by title and abstract. The remaining 33 articles were searched through full text; out of them, we were unable to retrieve 13 articles. Finally, a quality assessment for each article was done, and 11 studies with a score of greater than 70% were included in the review. There were six RCTs, three systematic reviews and meta-analyses, and two cohort studies. Figure 1 presents a flow diagram that illustrates the screening procedure and study selection [23].

Tables 3-5 demonstrate how each study was assessed using the appropriate quality evaluation tool for the respective study type [24-26].

**Table 3:** Review of the author’s summary of the risk of bias assessment of randomized control trial. H: high risk; U: unclear; L: low risk [24]

First author, Year	Random Sequence Generation	Allocation Concealment	Selective Reporting	Other Bias	Blinding of Participants and Personnel	Blinding of Outcome Assessment	Incomplete Outcome Data
Weinblatt et al., 1999 [12]	L	L	L	L	L	L	L
Bathon et al., 2006 [13]	L	L	L	L	L	L	L
van der Heijde et al., 2007 [27]	L	L	L	L	L	L	L
Emery et al., 2010 [28]	L	L	L	L	L	L	U
Moreland et al., 2012 [29]	L	L	L	U	L	L	L
Gallo et al., 2016 [30]	L	L	L	L	L	L	U

**Table 4:** Review the author’s summary of critical appraisal for systematic review and meta-analysis. Y: yes; PY: partial yes; N: no [26]. Domain 1: Population, intervention, comparison, and outcome (PICO) components included in research questions and inclusion criteria. Domain 2: Explicit statement that the review methods were established prior to the conduct of the review and justification of any significant deviations from the protocol. Domain 3: Explanation of the selection of the study designs for inclusion in the review. Domain 4: Use of a comprehensive literature search strategy. Domain 5: Study selection performed in duplicate. Domain 6: Data extraction performed in duplicate. Domain 7: A list of excluded studies and justifications for the exclusions. Domain 8: Description of the included studies in adequate detail. Domain 9: Satisfactory technique for assessing the risk of bias (RoB) in individual studies that were included in the review. Domain 10: Report on the sources of funding for the studies included in the review. Domain 11: Use appropriate methods for the statistical combination of results if meta-analysis is performed. Domain 12: Assessing the potential impact of RoB in individual studies on the results of the meta-analysis or other evidence synthesis. Domain 13: RoB in individual studies when interpreting or discussing the results of the review. Domain 14: Satisfactory explanation for and discussion of any heterogeneity observed in the results of the review. Domain 15: Adequate investigation of publication bias (small study bias) and discussion of its likely impact on the results of the review for quantitative synthesis. Domain 16: Potential sources of conflict of interest, including any funding received for conducting the review.

First Author, Year	Domain 1	Domain 2	Domain 3	Domain 4	Domain 5	Domain 6	Domain 7	Domain 8	Domain 9	Domain 10	Domain 11	Domain 12	Domain 13	Domain 14	Domain 15	Domain 16
Blumenauer et al., 2003 [6]	Y	PY	Y	PY	Y	Y	Y	Y	Y	Y	Y	Y	Y	Y	N	Y
Chen et al., 2016 [7]	Y	PY	Y	PY	N	Y	PY	Y	Y	N	Y	Y	Y	Y	N	Y
Wu et al., 2021 [31]	Y	PY	Y	PY	Y	Y	N	PY	PY	N	Y	Y	Y	N	Y	Y

**Table 5:** Review of the author’s summary of quality assessment of cohort studies. "1" point for each Domain and "2" points for comparability [25]. Domain 1: representativeness of the exposed cohort. Domain 2: Selecting the non-exposed cohort. Domain 3: Ascertainment of exposure. Domain 4: Demonstration that the outcome of interest was not present at the start of the study. Domain 5: Comparability of cohorts based on the design or analysis. Domain 6: Assessment of outcomes. Domain 7: Follow up long enough for outcomes to occur. Domain 8: Adequacy of follow-up of cohorts.

First Author, Year	Domain 1	Domain 2	Domain 3	Domain 4	Domain 5	Domain 6	Domain 7	Domain 8
Wassenberg et al., 2023 [32]	1	1	1	1	2	1	1	1
Pappas et al., 2023 [33]	1	1	1	1	2	1	1	1

Study characteristics and outcome

The main features of an individual study, with outcomes measured and a conclusion drawn, are demonstrated in chronological order in Table 6.

**Table 6:** Findings of accepted studies in the analysis. RCT: randomized controlled trial; ETN: etanercept; JAK inh: JAK inhibitor; MTX: methotrexate; RA: rheumatoid arthritis; QoL: quality of life; DMARD: disease-modifying anti-rheumatic medications; mTSS: modified Sharp score; ES: erosion score; JSN: joint narrowing score; ACR20, ACR50, or ACR70: American College of Rheumatology 20, 50, or 70; DAS 28: disease activity score 28; HAQ-DI: Health Assessment Questionnaire Disability Index; HAQ score: Health Assessment Questionnaire Score; bDMARD: biological disease-modifying anti-rheumatic medications; cDMARDs: conventional disease-modifying anti-rheumatic medications; TEMPO: trial of etanercept and methotrexate with radiographic and patient outcome; COMET: combination of methotrexate and etanercept trial

Study	Author	Year	Type of Study	Patients	Drug Studies	Purpose of Study	Outcome	Conclusion
1	Pappas et al [33]	2023	Cohort study	2967	ETN, Adalimumab, JAK inh	Effectiveness and persistence of ETN, adalimumab & JAK inh.	Disease activity and treatment persistence	No difference in clinical effectiveness and treatment persistence rates in bDMARD-naïve patients initiating ETN, adalimumab, or JAK inh either alone or in combination with cDMARDs.
2	Wassenberg et al [32]	2022	Cohort study	1821	ETN	Radiographic progression in RA treated with ETN for 36 months in Germany.	Modified Sharp score (mTSS), ES, joint narrowing score (JSN)	Lower radiographic progression with ETN.
3	Wu M. et al [31]	2021	Systematic review with Meta-analysis	6812	ETN, Anakinra & Abatacept	Efficacy of fusion protein combination in the treatment of RA.	ACR 20	ETN and MTX combination has the highest probability of optimal treatment as compared to other combinations.
4	Chen M et al [7]	2016	Systematic review and meta-analysis	3878	ETN vs MTX or Placebo	Efficacy of ETN	ACR 20,50 within 24 weeks & 1-3 years	ETN shows a higher rate of efficacy as compared to treatment by placebo or MTX. Higher dose of ETN might be more effective for active RA.
5	Gallo G et al [30]	2016	RCT	494	ETN, MTX	Effect of MTX dosage on clinical, functional, and QoL in RA when used in combination with ETN.	DAS 28, HAQ-DI. QoL	Regardless of MTX dosage, patients in the TEMPO and COMET trials who received both ETN and MTX exhibit similar efficacious outcomes at 24 months.
6	Moreland et al [29]	2014	RCT	755	ETN, MTX, Sulfasalazine, hydro chloroquine	To assess effectiveness of oral triple therapy versus ETN + MTX combination therapy.	DAS28. HAQ score. Radiographic result. Safety & tolerability	The radiographic advantage of etanercept with methotrexate was shown to be statistically significant, albeit clinically insignificant, in comparison to oral triple treatment. No difference in mean DAS28 and safety among the two groups.
7	Emery et al [28]	2010	RCT	398	ETN vs MTX	Evaluation of combination ETN+MTX on long-term remission and radiographic progression in early active RA.	DAS 28. Modified Sharp score	Early sustained combination ETN+MTX therapy seems superior to MTX monotherapy.
8	Heijde et al [27]	2007	RCT	682	ETN +MTX	3 year clinical & radiographic outcome & safety of ETN, MTX.	DAS28. ACR response. Radiographic outcome. Safety assessment	Combination therapy results in significant improvement in DAS & disease remission than monotherapy.
9	Bathon et al [13]	2006	RCT	5815	ETN vs MTX	Safety and efficacy of ETN in elderly and younger patients.	ACR 20,50. HAQ Score, Sharp Score	Significant Improvement in Disease activity and function without additional safety concerns.
10	Blumenauer et al [6]	2003	Systematic review and meta-analysis	2842	ETN + MTX	Benefit and harm of ETN+ DMARD as compared to DMARD monotherapy.	ACR50. Radiographic progression. Reduction in disability score. Adverse effect. Serious adverse effect. Serious infection	ETN+MTX combination therapy is more effective than ETN monotherapy.
11	Weinblatt et al [12]	1999	RCT	89	ETN, MTX	Determine whether addition of ETN to MTX provides benefit.	ACR 20,50. Adverse events	Addition of ETN to MTX results in rapid and sustained improvement.

Review

Discussion

RA is a chronic autoimmune disorder that causes significant joint swelling, pain, and a reduced QoL. Patients are at risk of developing progressive joint damage [27-33]. Genetics plays a major role in the etiology of RA. It is believed to be the outcome of the interaction between environmental influences, such as smoking, and the genotypes of the patients [34]. TNF is a crucial molecule that regulates the inflammatory alterations that take place in the RA synovium, despite the pathophysiology of the disease remaining unclear, according to Matsuno et al. [35]. TNF may accelerate tissue remodeling by stimulating the synthesis of matrix-degrading proteases, enhance cell migration by stimulating the synthesis of cellular adhesion molecules, and boost the production of pro-inflammatory cytokines [7]. Furthermore, TNF suppression may prevent or delay gradual joint degeneration [7].

Pharmacological and non-pharmacological treatments are both necessary for the management of RA patients. Currently, early therapy with disease-modifying anti-rheumatic medications is the mainstay of care [34]. Disease-modifying anti-rheumatic medications (DMARDs) have been demonstrated to improve QoL, lessen disease activity, and slow down joint deterioration. Although DMARDS constitute the cornerstone of treatment, many people cannot tolerate or do not react to standard DMARDS [27-33]. Biological agents (TNF inhibitors) became the second line of treatment for such patients. One of the biological agents, fusion protein, is a unique single protein with two partial functional capabilities that is created through the genetic fusion of two or more genes. The fusion protein's structure is divided into two halves, each of which performs a specific function and allows for molecular binding. The functional component, known as Fc, binds to particular receptors in order to attain pharmaceutical qualities [33-36]. ETN is a dimeric human TNF receptor (TNFR) p75-Fc fusion protein composed of two extracellular domains of the human 75 kDa (p75) TNFR connected by the constant Fc domain of human immunoglobulin one (IgG1) [36]. The dimeric structure of ETN not only improves its binding ability but also offers significantly more competitive TNF inhibition [36]. The aim of this study is to assess the efficacy and safety of ETN, either as monotherapy or in combination with MTX. Clinical effectiveness is measured by the 20%, 50%, and 70% improvement criteria of the American College of Rheumatology (ACR 20%, ACR 50%, and ACR 70%), a health assessment questionnaire (HAQ) to evaluate the reduction in disability, remission by disease activity score (DAS28), and radiographic progression by modified total Sharp score (mTSS). Safety measurements will include AEs and serious infections.

The advantages of ETN will be discussed in this section. We measured how well the ETN and MTX combination worked by looking at the ACR response rates. The rates for the ETN and MTX combination groups were higher than those for the ETN or MTX monotherapy groups [12-14]. A double-blinded RCT by Weinblatt [12] measures the effectiveness of ETN in RA patients who had an inadequate response to MTX in 24 weeks. In this study, 89 patients who had active RA despite a stable dose of MTX (15-25 mg) were given ETN (25 mg, SC twice weekly) or placebo. Seventy-one percent of people in the ETN+MTX group achieved ACR20 by 24 weeks, compared to 27% in the placebo and MTX groups (p<0.001), and 39% achieved ACR50 in the ETN+MTX group, compared to 3% in the placebo + MTX group (p<0.001). However, the dosage of ETN was also found to have an impact on the ACR response. For example, a meta-analysis by Chen et al. [7], who reviewed 12 studies with a total of 3878 patients measures the efficacy of ETA at various dosages for the treatment of active RA. ACR20, 50, and 70 showed an overall response rate of 1.10 (95% CI: 1.02-1.19, P < 0.02), 1.37 (95% CI: 0.98-1.92, P < 0.07), and 1.27 (95% CI: 1.02-1.58, P < 0.03) for 25 mg versus 10 mg ETA twice weekly. He finally concludes that a higher dose of ETN (25 mg twice weekly) had higher efficacy than a low dose (10 mg twice weekly) in the ACR 20 and ACR 70 responses, whereas no significant difference was noted in the ACR 50 response. Although various confounding variables, such as differences in ethnicity, age, duration of RA, and disease activity, may need to be considered for further analysis.

The DAS 28 score (DAS 1.6 and DAS 28 2.6) quantifies the degree of disease remission. The TEMPO study by van der Heijde et al. [27] was a double-blind RCT in which 682 patients were given either 25 mg of ETN twice a week, 20 mg or less of MTX once a week, or a mix of the two drugs. He found that more patients who were given both ETN and MTX had low disease activity (64.5% with DAS <2.4 and 56.3% with DAS 28 <3.2) than those who were only given one drug (44.4% with DAS <2.4 and 33.2% with DAS 28 <3.2 for ETN and 38.6% with DAS <2.4 and 28.5% with DAS 28 <3.2 for MTX, with P<0.01). However, another study( TEAR Study) by Moreland [29], which was a two-year double-blinded RCT, mentions that early combination therapy with either ETN and MTX or oral triple therapy at 24 months demonstrates a greater reduction in DAS 28-ESR compared to initial MTX therapy (DAS 28-ESR: 3.6 vs. 4.6, p<0.0001), but also shows no difference in mean DAS 28-ESR during 48-102 weeks in both groups.

We also analyze advantages by measuring the mTSS for the radiographic progression of RA. Although there were no statistically significant differences in radiographic progression, the ETN and MTX combination showed some clinical advantages over monotherapy or triple therapy. A study by Blumenauer et al. [6] mentions that, when compared to those taking DMARD, individuals getting ETN showed lesser changes from baseline in the TSS and ES scores at 12 months and two years, as well as a reduced change in the ES score at one year (with a trend of P value = 0.06 for the TSS score). The TEMPO study [27] found that people who were taking ETN along with DMARD stopped getting worse on X-rays. This was especially true for TSS and ES, where the negative mean scores show that the change from baseline had stopped. Wassenberg's PRERA study, a non-interventional cohort study that took place in Germany in 2022, also assessed the radiographic progression in RA patients who had been receiving ETN for at least 36 months [32]. There were significant differences in the intervals between the baseline X-ray and the first, second, and historic follow-up X-rays among the patients [36]. Annualized radiographic progression (mTSS, ES, and JSN) was generated to help the comparison. The study's first 18 months (phase one) saw a significantly decreased annualized radiographic progression in patients with available historical X-rays for RA compared to the pre-ETN treatment. The ES indicated that ETN treatment slowed joint degradation, and P-values for non-progression were extremely significant (p 0.005). A lower number of patients and a lack of ethnic variability may limit its application to the general population.

ETN is relatively better tolerated, but we also assess the harmful events experienced by patients receiving ETN. The only events that happened considerably more frequently in the ETN plus-MTX group were reactions at the injection site (42% vs. 7%, P<0.001) [12]. The fact that ETN users were far more likely to experience this specific side effect than those taking a placebo or DMARD may have contributed to the study's lack of blinding because participants may have realized what kind of treatment they were receiving. Overall, infection was the most frequent AE; upper respiratory infections accounted for around one-third of the illnesses [12]. No discernible difference in the incidence of additional AEs was seen at any point during the study when ETN monotherapy was compared to placebo or ETN + DMARD [6]. Several side events were significantly more likely to occur with DMARD alone when ETN monotherapy was compared to it [6]. During the third year of the TEMPO study [27], no new safety concerns were raised. Additionally, it states that in the combination, ETN, and MTX groups, 23.4%, 22.9%, and 18.9% of patients reported non-infectious serious AEs, while 7.4%, 6.4%, and 8.3% of patients reported serious infections. During the three years of the period, all major illnesses that happened more than once were: pneumonia, septic arthritis, skin infection, cellulitis/abscess, and postoperative wound infection. Out of 13 patients with a history of tuberculosis, only one experienced reactivated TB during the third year in the combination group. Over the course of the three-year study, five patients died. Congestive heart failure, autoimmune disorders, demyelinating diseases, and malignancy, specifically lymphomas are also some of the safety concerns that have emerged recently [36]. Seventy incidences of lymphoma were reported on 230,000 people exposed to ETN during the 2003 FDA study of the safety of TNF antagonists. This would suggest that the incidence of lymphoma is between two and three cases per 10,000 patient-years, with an estimated rate of three cases per 10,000 patient-years in the normal population [36-37]. Though the baseline characteristics of patients may vary among different studies, such as genetic makeup, ethnicity, and age, this would impact the final results. We need further long-term studies to justify the rate of significant AEs in patients receiving ETN.

Limitation

The papers included in this study were restricted to those written in English or where an English translation is available and published between 1999 and 2023. Some of the studies with different classes of medications were also included. We have excluded grey literature and biosimilars in this study. Very limited numbers of RCTs have been found in the last five years for the safety and efficacy of ETN. Moreover, we found that there are variations in baseline characteristics, such as differences in ethnicity, age, duration of RA, and disease activity, that may need to be considered for further analysis.

Furthermore, it was noted that the duration of follow-up varies among different studies, and no more than a three-year period was included. In order to measure safety over an extended period of time, long-term trials with a large number of participants needed to be conducted.

## Conclusions

In conclusion, combination therapy of ETN and MTX seems to be superior to monotherapy of ETN. ACR response and DAS score, which are tools to measure the clinical effectiveness of pharmacotherapy in patients with RA, seem to improve in patients who receive combination therapy of ETN and MTX as compared to those who receive monotherapy. Though radiographic progression shows no statistical significance, clinical improvement was found in RA patients. Higher doses of ETN have more favorable outcomes than lower doses. ETN, which is generally considered a well-tolerated drug, no indication of a difference in the short-term rates of major AEs has been discovered in this review, whether ETN is used alone or in combination with DMARDs. Reactions to ETN alone at the injection site were the most frequent side effects noted. On the other hand, there are worries about rising infection rates, especially for tuberculosis, and perhaps rising malignancy risks. Finally, our recommendation is therefore that more research in the form of RCTs or cohort studies is required, with a larger sample size, similar baseline properties among selected patients, and a longer follow-up period required to determine the benefits and long-term safety.
